# Secure multiparty computation of a comparison problem

**DOI:** 10.1186/s40064-016-3061-0

**Published:** 2016-09-05

**Authors:** Xin Liu, Shundong Li, Jian Liu, Xiubo Chen, Gang Xu

**Affiliations:** 1 School of Computer Science, Shaanxi Normal University, Xi’an, 710062 China; 2 School of Information Engineering, Inner Mongolia University of Science and Technology, Baotou, 014010 China; 3 School of Communication and Information Engineering, Nanjing University of Posts and Telecommunications, Nanjing, 210003 China; 4 Information Security Center, State Key Laboratory of Networking and Switching Technology, Beijing University of Posts and Telecommunications, Beijing, 100876 China; 5 School of Software Engineering, Beijing University of Posts and Telecommunications, Beijing, 100876 China

**Keywords:** Secure multiparty computation, Comparison problem, Vector encoding method, GM encryption scheme

## Abstract

Private comparison is fundamental to secure multiparty computation. In this study, we propose novel protocols to privately determine $x>y, x<y$, or $x=y$ in one execution. First, a 0–1-vector encoding method is introduced to encode a number into a vector, and the Goldwasser–Micali encryption scheme is used to compare integers privately. Then, we propose a protocol by using a geometric method to compare rational numbers privately, and the protocol is information-theoretical secure. Using the simulation paradigm, we prove the privacy-preserving property of our protocols in the semi-honest model. The complexity analysis shows that our protocols are more efficient than previous solutions.

## Background

The Millionaires’ Problem is first proposed by Yao (1982). The problem is described as follows: Alice and Bob have their own wealth *x* and *y* million, respectively; they want to know who is richer without disclosing their wealth. The Millionaires’ Problem is abstracted as *Greater Than* or *GT* problem.

The GT problem has been developed into secure multiparty computation (SMC). The SMC studies the following problems: two or more parties want to jointly compute a function *f*. In these situations, the parties get correct results, but do not disclose their own inputs to others. Goldreich et al. (1987) proposed a general theoretical solution to all SMC problems using the circuit evaluation and defined the SMC security (Goldreich 2004). However, using the general SMC solution to all problems is impractical for efficiency reason. So Golidreich further pointed that we should study specific solutions to different problems in practice. In addition, Goldwasser (1997) predicted that SMC, which was a powerful tool and had rich theoretical basis but whose real-life usage was only beginning, would become an integral part of our computing reality in the future.

Motivated by the prediction, researchers have studied many specific SMC solutions, including private sorting (Liu et al. 2012), private determining the relationship of sets (Dachman-Soled et al. 2012), private computional geometry (Shundong et al. 2014), private voting (Toft 2011), and private data mining (Bogdanov et al. 2012; Fu et al. 2015b) etc.

At present, SMC protocols are studied in either the semi-honest model or the malicious model, and proposing a SMC protocol in the malicious model is more difficult than in the semi-honest model. However, Goldreich designed an important compiler. Given a protocol $\pi$ that privately computes a function *f* in the semi-honest model, his compiler can produce a new protocol $\pi '$ that privately computes *f* in the malicious model. In addition, some SMC problems have not been efficiently solved and some SMC problems are not solved even in the semi-honest model (Gu et al. 2015; Xia et al. 2015; Pan et al. 2015; Ren et al. 2015). So we propose our protocols in the semi-honest model.

The GT problem is a building block of many SMC protocols (Shim 2012; Zhang et al. 2011; Banu and Nagaveni 2013; Lin et al. 2014; Fu et al. 2015a; Hong and Sun 2016). Cryptographic researchers have proposed some GT protocols. Cachin (1999) proposed a GT protocol based on the $\phi$-hiding assumption, but this protocol need a trusted third party. Ioannidis and Grama (2003) used the oblivious transfer (*OT*) scheme to construct a GT protocol, but the length of inputs was restricted by a secure parameter of the *OT* scheme. Fischlin (2001) used the Goldwasser–Micali encryption scheme to construct a two-round GT protocol, and its computation cost is ($\lambda d\text {log} N+6d\lambda +3d$) modular multiplications (*d* is the length of private inputs, $\lambda$ is set to 40–50).

Later, Li et al. (2005) constructed a function *F* to compare two function values instead of plaintexts, and used the $OT_m^1$ scheme to compare any data. Schoenmakers et al. (2004) used a threshold homomorphic encryption scheme to solve the GT problem, in which inputs was shared among a group of parties. The communication cost was *O*(*n*). Blake and Kolesnikov (2004) used the Paillier encryption schemem to construct a two-round GT protocol whose computation cost was $O(n \text {log} N)$ modular multiplications. Lin and Tzeng (2005) proposed a two-round GT protocol using the ElGamal multiplicatively homomorphic encryption scheme and a 0–1 encoding method, and the computation cost was $O(n\text {log}\ p)$ modular multiplications. Grigoriev and Shpilrain (2014) used a public encryption scheme to solve the Millionaires’ Problem with two-round communications and computation costs is $(6\text {log}p+3d)$ modular multiplications. Maitra et al. (2015) proposed a two-round protocol to solve the Millionaires’ Problem with computation costs of $(2d\text {log}p)$ modular multiplications.

However, some previous GT solutions just compare integers, some of them cannot determine $x>y, x<y$, or $x=y$ in one execution, some of them need a trusted third party, and some of them are inefficient.

In this study, we propose new solutions to the GT problem. We introduce a 0–1-vector encoding method, and use the Goldwasser–Micali (abstracted as *GM*) encryption scheme to compare integers efficiently. Then we present a protocol to privately compare rational numbers in one execution by computing the area $S_{\triangle }$ of a triangle.

**Our contribution:**

1. We introduce a 0–1-vector encoding method which is used to encode a number into a vector. Using the encoding method, we can transform the comparison problem into a vector-element-selecting problem. This method is more efficient than directly comparing two numbers.
2. We propose a private comparison protocol for integers based on the XOR homomorphism of the GM encryption scheme and the vector encoding method. Its computation cost for a vector of length *L* is ($6L+4$) modular multiplications and the communication cost is two rounds at most.
3. Further, we use a geometric method to privately compare two rational numbers. By privately computing the sign of a triangle area $S_{\triangle }$, we determine whether $x=y, x<y$, or $x>y$ in one execution. The protocol just needs five additions and eight multiplications, so its computation cost can be neglected and its communication cost is one round. The protocol is information-theoretical secure.

The rest of this paper is organized as follows:

“Related work” section introduces related definitions and methods, including the ideal SMC model, the semi-honest model, the simulation paradigm, the Goldwasser–Micali encryption scheme, the 0–1-vector encoding method, and the secure computation method of the area of a triangle; “New protocols to privately solve a comparison problem” section proposes new protocols for comparing integers and rational numbers, shows the correctness and security analysis of our protocols, and proves their privacy-preserving property using the simulation paradigm; “Complexity analysis” section compares the computational and communication complexity of our protocols with previous solutions; “Conclusion” section concludes this work.

## Related work

### Ideal SMC model

The ideal SMC model is the simplest SMC model. It needs a trusted third party (TTP), who always tells the truth, never lies, and never discloses any input information. So the ideal SMC protocol is the most secure. If such a TTP exists, Alice (holding *x* ) and Bob (holding *y* ) can privately compute *f*(*x*, *y*) as follows:

1. Alice sends *x* to TTP;
2. Bob sends *y* to TTP;
3. TTP computes $$f(x,y)=(f_1(x,y),f_2(x,y))$$;
4. TTP sends the result to Alice and Bob.

Theoretically, the above protocol can solve any SMC problems, but the TTP cannot be easily found in practice. So we need to study SMC protocols without TTP.

### Semi-honest model

We assume that all parties are semi-honest. A semi-honest party truthfully follows a protocol and sends correct inputs to others, except that he may record all intermediate computation and try to derive other parties’ private inputs from the record. Goldreich has proved that, a protocol which can privately compute a functionality *f* in the semi-honest model can be complied, by introducing a bit commitment macro, into another protocol which can compute the functionality *f* in the malicious model. The semi-honest model is not only an important methodological tool but may also provide a good model in many settings. It suffices to prove that a protocol is secure in the semi-honest model.

If the information that a party efficiently computes from the execution of a protocol can also be efficiently computed on its input and output, the protocol is private. This intuition is formalized by the simulation paradigm. That is, a party’s *view* in a protocol execution can be simulated by its input and output. If so, the parties learn nothing from the protocol execution itself, and the protocol is private. Notations and definition are following:

**Notations:** Alice holds *x*, and Bob holds *y* in a two-party SMC protocol.

1. Alice and Bob’s inputs are *x*, *y*, respectively;
2. They propose a protocol $\pi$ to compute a function *f*, where *f* is a probabilistic polynomial time functionality;
3. Alice and Bob obtain message sequences $view_1^\pi (x,y)=(x,r^1,m_1^1, \ldots , m_t^1)$ and $view_2^\pi (x,y)=(x,r^2,m_1^2,\ldots , m_t^2)$, respectively, where $r^1$ or $r^2$ is the result of her or his internal coin toss, and $m_i^1$ or $m_i^2$ is her or his received message;
4. Alice’s output is $output_1^\pi (x,y)$, and Bob’s output is $output_2^\pi (x,y)$.

#### **Definition 1**

For a function $f, \pi$ privately computes *f* if there exists a probabilistic polynomial time algorithm, denoted by simulators $S_1$ and $S_2$, such that:

1 $$\left\{ (S_1(x,f_1(x,y)),f_2(x,y))\right\} _{x, y} \mathop {\equiv }\limits ^{c} \left\{ (view_1^\pi (x,y),output_2^\pi (x,y))\right\} _{x, y}$$

2 $$\left\{ (f_1(x,y),S_2(y,f_2(x,y))\right\} _{x, y} \mathop {\equiv }\limits ^{c} \left\{ (output_1^\pi (x,y),view_2^\pi (x,y))\right\} _{x, y}$$

where $\mathop {\equiv }\limits ^{c}$ denotes computational indistinguishability.

To prove that a multiparty computation protocol is private, we must construct the simulators $S_1$ and $S_2$ such that (1) and (2) hold.

### Goldwasser–Micali public key cryptosystem

A multiplicative group of $Z_n$ is $Z_n^*=\{x\in Z_n | gcd(x, n) = 1\}$. Let $a\in Z_n^*$. *a* is called a quadratic residue modulo *n* if there exists an $x\in Z_n^*$ such that $x^2\equiv a(\bmod n)$. If no such *x* exists, *a* is called a quadratic non-residue modulo *n*. For any $r\in Z_n^*, r^2 \bmod n$ is always a quadratic residue modulo *n*. The Goldwasser–Micali (GM) public key cryptosystem (Goldwasser and Micali 1984) is the first probabilistic cryptosystem based on the fact that if *t* is quadratic nonresidue, then so is $tr^2$ for any $r\in Z_n^*$, and which consists of following three algorithms:

**Key generation:** Takes a security parameter *k* as an input. The GM encryption scheme chooses two *k*-bit primes *p* and *q*, sets $n = pq$, and picks a $t \in Z_n^1$ ($Z_n^1$ is the subset of $Z_n^*$ containing the elements with Jacobi symbol) such that *t* is a quadratic nonresidue modulo *n*. It then publishes (*n*, *t*) as public keys, and keeps the private keys (*p*, *q*) secret.

**Encrypt:** Takes a message $m \in \{0,1\}$ as input, the public key $\{n, t\}$, and a random number *r*. It encrypts $m_i$ as follows:

$$E(m_i)=t^{m_i} r_i^2 \bmod n=\left\{ \begin{array}{ll}&tr_i^2\bmod n, \quad m_i=1;\\ &r_i^2 \bmod n, \quad m_i=0 \end{array} \right.$$

**Decrypt:** Based on the private key (*p*, *q*), it decrypts $E(m_i)$ as follows:

$$m_i=\left\{ \begin{aligned}&0, \quad \left( \frac{E(m_i)}{p}\right) =\left( \frac{E(m_i)}{q}\right) =1;\\&1, \quad \left( \frac{E(m_i)}{p}\right) =\left( \frac{E(m_i)}{q}\right) =-1 \end{aligned} \right.$$

where $(\frac{a}{p})$ is the Legendre symbol, which is defined as follows:

$$\left( \frac{a}{p}\right) =\left\{ \begin{aligned}&\quad 1, \quad (p \nmid a, \,<a>_p is\,\,quadratic\,\,residue\,\,modulo);\\&-1, \quad (p \nmid a, \,<a>_p \,is\,\,quadratic\,\,non{-}residue\,\,modulo);\\&\quad 0, \quad (p | a). \end{aligned} \right.$$

**Homomorphism:**

The GM encryption scheme has homomorphism, that is:

$$E(m_i)\cdot E(m_j)=\left\{ \begin{array}{ll} r_i^2r_j^2 \bmod n, &\quad m_i=0, m_j=0;\\ tr_i^2r_j^2 \bmod n, &\quad m_i=0, m_j=1;\\ t^2r_i^2r_j^2 \bmod n, & \quad m_i=1, m_j=1;\\ tr_i^2r_j^2 \bmod n, &\quad m_i=1, m_j=0. \end{array} \right.$$

From the above observation, it shows that $E(m_i)\cdot E(m_j)=E(m_i \oplus m_j)$ and the plaintexts $m_i \in \{0,1\}$, so the GM encryption has XOR homomorphism.

### Vector encoding method

In this subsection, we introduce a vector encoding method. The vector encoding method can encode a natural number *k* into a vector *v* as follows:

The vector of a number *k* is encoded as follows:

3 $$v=\{v_1,v_2, \ldots ,v_n\},$$

where $$v_{i}=\left\{ \begin{array}{ll}&\alpha ,\quad 1\le i< k;\\&\beta ,\quad i\ge k \end{array} \right., \quad \alpha \ne \beta$$.

### Privately computing the area of a triangle

Li et al. (2010) have proposed a SMC protocol of computing the area of a triangle, as follows.

Suppose that there is a triangle $\triangle P_0P_1P_2$ with three vertices $P_0(x_0, y_0), P_1(x_1, y_1), P_2(x_2, y_2)$, the area of $\triangle P_0P_1P_2$ is computed without security requirements as follows:

4 $$S_{\triangle P_0P_1P_2}= \frac{1}{2} \left| \begin{array}{clr} x_0 &{} y_0 &{} 1 \\ x_1 &{} y_1 &{} 1 \\ x_2 &{} y_2 &{} 1 \end{array} \right| = \frac{1}{2}[x_0(y_1-y_2)-x_1(y_0-y_2)+x_2(y_0-y_1)],$$

where the sign of $S_{\triangle P_0P_1P_2}$ is positive if and only if $(P_0\rightarrow P_1\rightarrow P_2\rightarrow P_0)$ form a counterclockwise cycle, and negative if and only if $(P_0\rightarrow P_1\rightarrow P_2\rightarrow P_0)$ form a clockwise cycle.

The Formula (4) can be rearranged as follows:

5 $$S_{\triangle P_0P_1P_2}=\frac{1}{2}[x_0(y_1-y_2)+y_0(x_2-x_1)+(x_1y_2-x_2y_1)].$$

Let $$a=(y_1-y_2), b=(x_2-x_1), c=x_1y_2-x_2y_1$$, so

6 $$S_{\triangle P_0P_1P_2}=\frac{1}{2}(ax_0+by_0+c)$$

By Formula (6), we can privately compute the sign of $S_ {\triangle P_0P_1P_2}$.

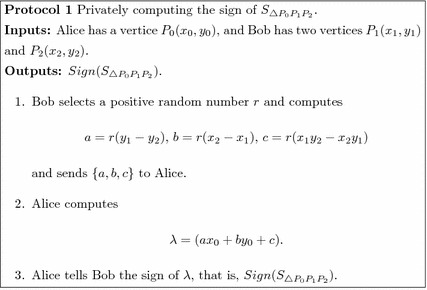



**Correctness and security:**

1. In the protocol, Alice knows $r(y_1-y_2)=a$ and $r(x_2-x_1)=b$. If $r, (y_1-y_2), (x_2-x_1)$ are integers and $\text {gcd}(x_2-x_1, y_1-y_2)=1$, Alice can compute *r* by $r=\text {gcd}(a,b)$. To avoid this situation, *r* should be selected by the form $l.2^i5^j$ ($i, j, l \in Z$), such as 5.425, 17.8125 or their multiple (Li et al. 2010).
2. In the protocol, Alice may get the slope *k* of a line $L_{P_1P_2}$ by computing $k=\frac{a}{b}$, but she cannot determine which line with the slope *k* and cannot obtain $x_1, x_2, y_1$ and $y_2$, because there are three equations with five unknown variables. For Bob, the protocol is secure.
3. By the result, Bob just obtains $Sign (S_{\triangle P_0P_1P_2})$, and cannot compute $x_0$ and $y_0$. For Alice, the protocol is secure.

#### **Theorem 1**

*Protocol 1 is private*.

The conclusion is proved by showing two simulators $S_1$ and $S_2$ such that formulas (1) and (2) hold.

#### *Proof*

We first construct $S_1$ to simulate Alice’s computation. In view of $\{a, b, c\}$ and the slope $k=\frac{a}{b}, S_1$ selects two points $P_1'(x_1', y_1'), P_2'(x_2', y_2')$ and a random number $r'$ that satisfy $a'=r'(y_1'-y_2'), b'=r'(x_2'-x_1'), c'=r'(x_1'y_2'-x_2'y_1')$. $S_1$ computes

$$\lambda '=(a'x_0+b'y_0+c').$$

Note that in this protocol

$$view_1^\pi (P_0, (P_1', P_2'))=\{P_0, a, b, c, Sign(\lambda )\}, Sign(\lambda )=Sign(\lambda '),$$

$$f_1(P_0,(P_1, P_2))=f_2(P_0,(P_1,P_2))=output_1^\pi (P_0,(P_1, P_2))=output_2^\pi (P_0,(P_1, P_2))$$.

Let

$$S_1(P_0, f_1(P_0,(P_1, P_2))=\{P_0, a', b', c', Sign(\lambda ')\}.$$

Since $(x_1, y_1), (x_2, y_2)$ and $(x_1', y_1'), (x_2', y_2')$ are arbitrary points on a plane, they are computationally indistinguishable. The results obtained by applying deterministic computation to computationally indistinguishable objects are still computationally indistinguishable. Therefore, $\{a', b', c'\}$ and $\{a, b, c\}$ are computationally indistinguishable. Therefore,

$$\begin{aligned}& \{(S_1(P_0, f_1(P_0, (P_1, P_2))), f_2(P_0, (P_1, P_2)))\}\\ &\quad \mathop {\equiv }\limits ^{c} \,\{(view_1^\pi (P_0, (P_1, P_2)), output_2^\pi (P_0, (P_1, P_2)))\}.\end{aligned}$$

Now, we construct $S_2$. In view of $P_1, P_2$ and $Sign(S_{\triangle P_0P_1P_2}), S_2$ selects a point $P_0'(x_0', x_1')$ and simulates as follows:

1. $$S_2$$ computes

$$a=r(y_1-y_2),\quad b=r(x_2-x_1), \quad c=r(x_1y_2-x_2y_1).$$

2. $$S_2$$ computes

$${\lambda ''}=\left( ax_0'+by_0'+c\right) .$$

3. Bob knows the sign of ${\triangle P_0'P_1P_2}$, that is, $Sign(S_{\triangle P_0'P_1P_2})$.

Since $P_0(x_0, y_0)$ and $P_0'(x_0', y_0')$ are two arbitrary points that satisfy

$$Sign\left( S_{\triangle P_0P_1P_2}\right) =Sign\left( S_{\triangle P_0'P_1P_2}\right),$$

these two points are computationally indistinguishable. Note that in the protocol

$$view_2^\pi \left( P_0, (P_2, P_2)\right) =\left\{ (P_1, P_2), a,b, c, Sign\left( S_{\triangle P_0P_1P_2}\right) \right\} .$$

Let

$$S_2\left( (P_1, P_2), f_2(P_0, (P_1, P_2))\right) =\left\{ P_1, P_2, a, b, c, Sign\left( S_{\triangle P_0'P_1P_2}\right) \right\} .$$

By the method we choose $P_0'(x_0', y_0')$, and it must hold that $Sign(S_{\triangle P_0'P_1P_2})=Sign(S_{\triangle P_0P_1P_2})$, therefore $view_2^\pi (P_0, (P_1, P_2))$ and $S_2((P_1, P_2), f_2(P_0, (P_1, P_2))$ are computationally indistinguishable. It follows that

$$\begin{aligned}&\left\{ \left( f_1(P_0, (P_1, P_2)), S_2(P_0, f_2(P_0, (P_1, P_2)))\right) \right\} \\ &\quad\mathop {\equiv }\limits ^{c} \left\{ (output_1^\pi (P_0, (P_1, P_2)), view_2^\pi (P_0, (P_1, P_2)))\right\} .\end{aligned}$$

This completes the proof.

## New protocols to privately solve a comparison problem

In this work, we propose new protocols to solve the private comparison problem for integers and rational numbers. For the integer comparison problem, we use a 0–1-vector encoding method and the GM encryption scheme. For the rational numbers comparison problem, we use the method for computing the area of a triangle to determine the relationship of *x* and *y* in one execution privately. We analyze the correctness and security of our protocols, and prove their privacy-preserving property using the simulation paradigm.

### Privately solving a comparison problem for integers

Alice and Bob hold their own numbers *x*, *y*, and they do not want to disclose their numbers when they execute the protocol. Alice uses the 0–1-vector encoding method to map *x* into a vector *X* and encrypts *X* by the GM encryption scheme. Bob selects an element from the ciphertexts of the vector *X* and encrypts the element using the homomorphism of the GM encryption scheme. Alice decrypts the ciphertexts and knows $x>y, x<y$, or $x=y$.

We first present Protocol 2 to determine the relationship *P*(*x*, *y*) :  $x>y$ or $x\le y$. If we need to further determine $x< y$ or $x=y$, we use Protocol 3 to solve the comparison problem.

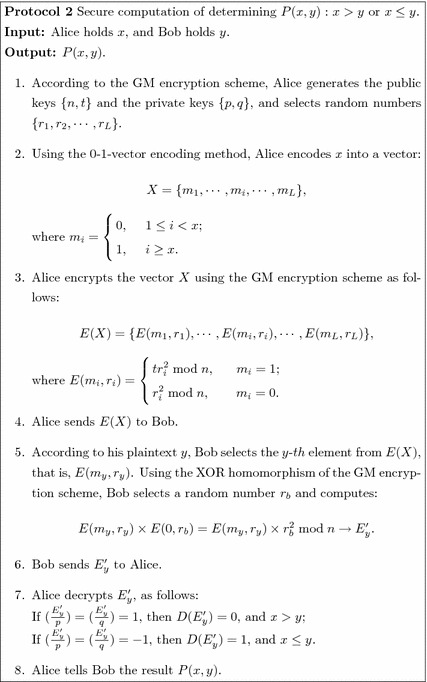



If the result is $x\le y$, we can use Protocol 3 to determine $x<y$ or $x=y$.

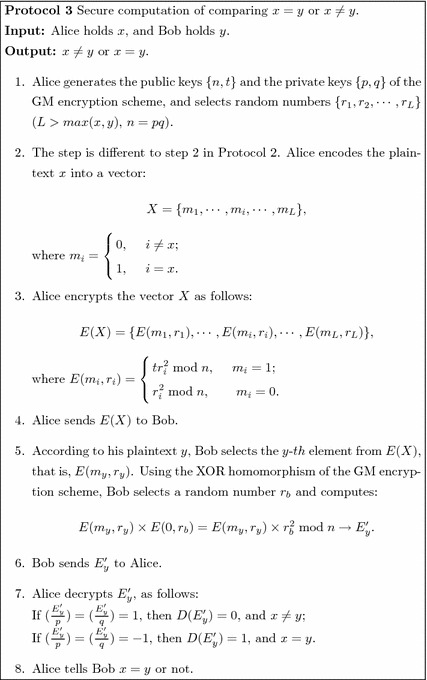



**Correctness and security:**

1. In Protocol 2 and Protocol 3, Step 5 is based on the XOR homomorphism of the GM encryption scheme, that is,
  $$E(m_y, r_y)\times E(0, r_b)=E(m_y, r_y)\times r_b^2 \bmod n=E(m_y \oplus 0);$$
  If $m_y=0, E(m_y, r_y)=r_y^2\bmod n$, then $D(E(m_y, r_y)\times r_b^2 \bmod n)=0$, so $x>y$ in Protocol 2 or $x\ne y$ in Protocol 3; If $m_y=1, E(m_y, r_y)=tr_y^2\bmod n$, then $D(E(m_y, r_y)\times r_b^2 \bmod n)=1$, so $x\le y$ in Protocol 2 or $x=y$ in Protocol 3;
2. Because the GM encryption scheme is a probabilistic encryption scheme, the same plaintext $m_i$ can be encrypted to different ciphertexts $E(m_i,r_i)$. Therefore, Bob does not discover the law of $E(m_i,r_i)$;
3. Alice’s random numbers $r_i$ and Bob’s random number $r_b$ are private. Bob cannot compute $E(m_i,r_i)$, and Alice cannot compute $E(0, r_b)$;
4. Bob selects the ciphertext $E(m_y,r_y)$, and encrypts $E(m_y,r_y)$, so Alice does not know which element Bob selects;
5. The prime numbers *p* and *q* are private, so Bob cannot decrypt *E*(*X*).

#### **Theorem 2**

*Protocol 2 is private*.

#### *Proof*

We will prove it by constructing $S_1$ and $S_2$ such that Formula(1) and (2) hold. $S_1$ works as follows:

1. The inputs are $\{x,P(x,y)\}$. $S_1$ randomly selects a number $y'$ such that $P(x,y)=P(x,y')$. $S_1$ uses $(x,y')$ to simulate the process. $S_1$ constructs the vector $X=\{m_1,m_2,\ldots , m_L\}$.
2. By the GM encryption scheme, $S_1$ encrypts *X* using different random numbers $r_i, E(X)=(E(m_1,r_1),E(m_2,r_2),\ldots , E(m_L,r_L))$;
3. $S_1$ selects a random $r'$, and computes $E(m_{y'},r_{y'})\times r'^2\bmod n\rightarrow E'(y')$;
4. $$S_1$$ decrypts $$D(E'(y'))\longrightarrow P(x, y')$$.

In the protocol, $view_1^\pi (x,y)=\{X,E(X),E_y',P(x,y)\}$.

Let

$$\{S_1(x,P(x,y))\}=\{X,E(X),E'(y'),P(x,y')\}.$$

Because $$P(x,y)=P(x,y'), E_y' \mathop {\equiv }\limits ^{c} E'(y')$$, therefore,

$$\{(S_1(x,P(x,y)), P(x,y))\}_{x,y}\mathop {\equiv }\limits ^{c} \{(view_1^\pi (x,y),output_2^\pi (x,y))\}_{x,y}.$$

Using the same method, we can construct $S_2$, such that:

$$\{(P(x,y),S_2(y,P(x,y)))\}_{x,y}\mathop {\equiv }\limits ^{c} \{(output_1^\pi (x,y),view_2^\pi (x,y))\}_{x,y}.$$

This completes the proof.

#### **Theorem 3**

*Protocol 3 is private*.

The proving process is similar to Theorem 2, so we omit the proof.

### Privately solving a comparison problem for rational numbers

In practice, most numbers need to be compared are rational numbers. The above protocols cannot compare rational numbers, so we propose a solution to compare rational numbers.

By “Privately computing the area of a triangle” section, we use two rational numbers *m* and *n* to construct three vertices of a triangle, and privately compute the sign of the area $S_\bigtriangleup$ to determine $m=n, m>n$, or $m<n$ in one execution.

**Fig. 1:**
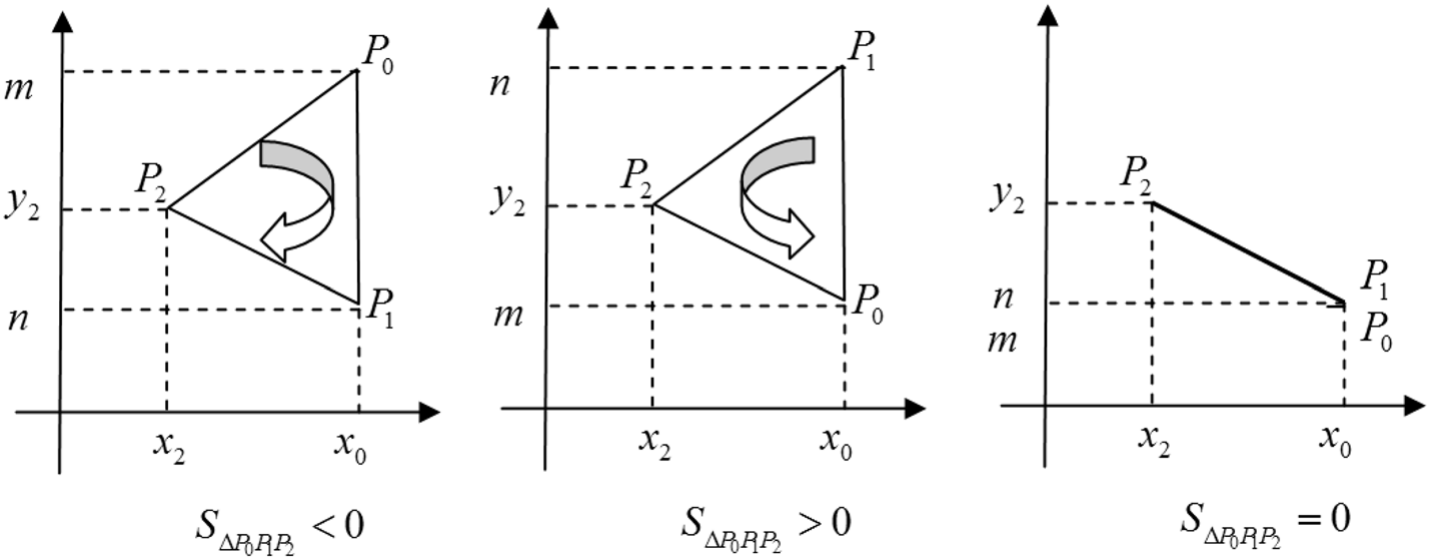
$$\bigtriangleup P_0P_1P_2$$

Alice and Bob agree on selecting a number $x_0$ as their abscissa. Alice constructs a point $P_0(x_0,m)$, and Bob constructs a point $P_1(x_0,n)$. Bob selects another point $P_2(x_2, y_2)$. $P_0, P_1$ and $P_2$ form a triangle. They invoke Protocol 1 to compute the sign of $S_{\bigtriangleup P_0P_1P_2}$, and judge whether $P_0$ on the top of $P_1$ or not. The result tells them $m>n, m=n$, or $m<n$, as follows in Fig. 1.

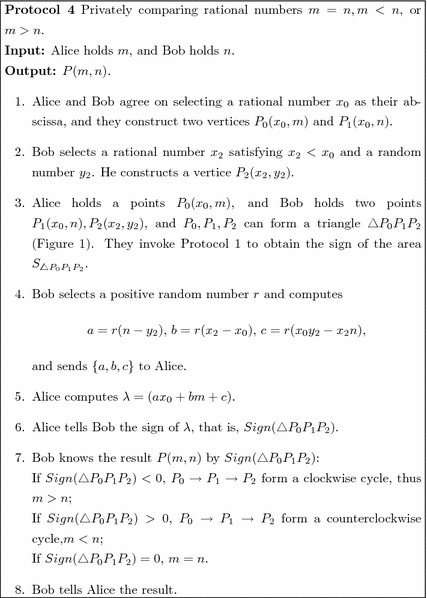



**Correctness and security:**

**Fig. 2:**
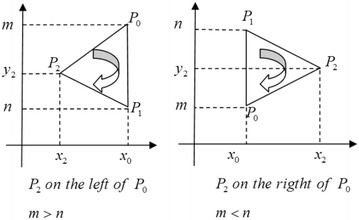
Example ($$\lambda <0$$)

1. In the protocol, Alice knows $r(n-y_2)=a$ and $r(x_2-x_0)=b$. If $r, (n-y_2), (x_2-x_0)$ are integers and $\text {gcd}(x_2-x_0, n-y_2)=1$, Alice can compute *r* by $r=\text {gcd}(a,b)$. But in Protocol 4, $x_0, x_2, y_2, n, a, b$ are rational numbers, thus Alice cannot compute *r* by $r=\text {gcd}(a,b)$.
2. In the protocol, Alice can get $\{a, b, c\}$, but there are three equations with four unknown variants and Alice cannot obtain $\{n, r, x_2, y_2\}$.
3. In step 6, Alice just computes $\lambda$, and she knows the sign of $S_{\Delta P_0 P_1 P_2 }$. Thus she knows $P_0 \rightarrow P_1 \rightarrow P_2$ is clockwise or counterclockwise, but she does not know whether $P_2$ is on the left or right of $P_0$, so she cannot know $m>n$ or $m<n$ (Fig. 2). Alice knows the sign of $S_{\Delta P_0 P_1 P_2 }$ is negative, and further knows $P_0 \rightarrow P_1 \rightarrow P_2$ is clockwise. But she does not know $m>n$ or $m<n$.
4. By the result, Bob just obtains $Sign (\triangle P_0P_1P_2)$, but cannot compute $x_0$ and *m*. For Alice, the protocol is secure.
5. The protocol does not use any public key encryption scheme, so it is information-theoretical secure.

#### **Theorem 4**

*Protocol 4 is private*.

The conclusion is proved by showing two simulators $S_1$ and $S_2$ such that Formulas (1) and (2) hold.

#### *Proof*

**Fig. 3:**
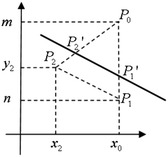
Selecting $$P_1', P_2'$$

In view of $\{a, b, c\}$ and the slope $k=\frac{a}{b}, S_1$ selects two points $P_1'(x_0, y_1'), P_2'(x_2', y_2')$ from any line with the slope *k* (Fig. 3), a random number $r'$, and computes $a'=r'(y_1'-y_2'), b'=r'(x_2'-x_0), c'=r'(x_0y_2'-x_2'y_1'), \lambda '=(a'x_0+b'm+c')$.

Note that in the protocol

$$view_1^\pi (P_0, (P_1, P_2))=\{P_0, a, b, c, \lambda \},$$

$$f_1(P_0,(P_1 P_2))=f_2(P_0,(P_1,P_2))=output_1^\pi (P_0,(P_1, P_2))=output_2^\pi (P_0,(P_1, P_2))$$.

Let $S_1(P_0, f_1(P_0,(P_1, P_2))=\{P_0, a', b', c', \lambda '\}$. Since $(x_0, n), (x_2, y_2)$ and $(x_0, y_1'), (x_2', y_2')$ are arbitrary points on the plane, they are computationally indistinguishable. The results obtained by applying deterministic computation to computationally indistinguishable objects are still computationally indistinguishable. Therefore, $\{a', b', c'\}$ and $\{a, b, c\}$ are computationally indistinguishable.

$$\begin{aligned}&\{(S_1(P_0, f_1(P_0, (P_1, P_2))), f_2(P_0, (P_1, P_2)))\} \\ &\quad\mathop {\equiv }\limits ^{c} \{(view_1^\pi (P_0, (P_1, P_2)), output_2^\pi (P_0, (P_1, P_2)))\}.\end{aligned}$$

**Fig. 4:**
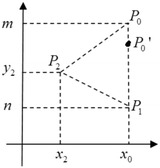
Selecting $$P_0'$$

Now, we construct $S_2$. In view of $P_1, P_2$ and $Sign(\triangle P_0P_1P_2), S_2$ selects a point $P_0'(x_0, m')$ (Fig. 4) and simulates as follows:

1. $$S_2$$ computes

$$a=r(n-y_2), b=r(x_2-x_0), c=r(x_0y_2-x_2n).$$

2. $$S_2$$ computes

$$\lambda '=(ax_0+bm'+c).$$

3. Bob knows the sign of $\lambda '$, that is, $Sign(\triangle P_0'P_1P_2)$.

Since $P_0(x_0, m)$ and $P_0'(x_0, m')$ are two arbitrary points that satisfy

$$Sign(\triangle P_0P_1P_2) =Sign(\triangle P_0'P_1P_2),$$

the two points are computationally indistinguishable.

Note that in the protocol

$$view_2^\pi (P_0, (P_1, P_2))=\{(P_1, P_2), a,b, c, Sign(\triangle P_0P_1P_2)\}.$$

Let

$$S_2((P_1, P_2), f_2(P_0, (P_1, P_2)))=\{P_1, P_2, a, b, c, Sign(\triangle P_0'P_1P_2)\}.$$

By the way, we choose $P_0'(x_0, m')$, and it must hold that $Sign(\triangle P_0'P_1P_2)=Sign(\triangle P_0P_1P_2)$. Therefore, $view_2^\pi (P_0, (P_1, P_2))$ and $S_2((P_1, P_2), f_2(P_0, (P_1, P_2))$ are computationally indistinguishable.

It follows that

$$\{(f_1(P_0, (P_1, P_2)), S_2(P_0, f_2(P_0, (P_1, P_2))))\}\mathop {\equiv }\limits ^{c} \{(output_1^\pi (P_0, (P_1, P_2)), view_2^\pi (P_0, (P_1, P_2)))\}.$$

This completes the proof.

## Complexity analysis

In the work, we compare the computational and communication complexity with previous solutions for secure computation of the comparison problem.

### Communication complexity

A protocol’s communication cost is usually measured in round. Yao’s protocol (Yao 1982) solves the GT problem with two rounds, but cannot determine whether $x=y$ or $x \ne y$. Cachin (1999) proposes a GT protocol depending on a trusted third party, and its communication cost is three rounds. Fischlin (2001) uses the GM encryption scheme to solve $x<y$ or $x\ge y$ with two-round communication cost. Ioannidis and Grama (2003) uses the $OT_2^1$ scheme to solve the GT problem, and its communication cost is *d* rounds, where *d* is the length of the private inputs. Blake and Kolesnikov (2004) uses the Paillier encryption scheme to solve $x>y, x<y$ or $x=y$, and its communication cost is two rounds. Lin’s protocol (Lin and Tzeng 2005) needs two-round communications based on the Elgamal encryption scheme. Grigoriev and Shpilrain (2014) propose a solution to Yao’s Millionaires’ problem based on a public encryption scheme and their communication cost is two rounds. Maitra et al. (2015) propose a unified approach to Millionaires Problem with rational players, and the solution needs two-round communications.

In our Protocol 2, we need one round to determine $x>y$ or $x\le y$. If we further determine $x<y$ or $x=y$, we also need one round communication by Protocol 3. Therefore, for the integer comparison problem, we need two-round communication cost at most.

In our Protocol 4, we determine $x<y, x>y$ or $x=y$ in one execution, so the communication cost is one round.

### Computational complexity

We use the number of modular multiplication to measure the computation costs of a protocol. The computation cost of Yao’s protocol (Yao 1982) is exponential, and it is impractical if inputs are very long. Fischlin (2001) uses the GM encryption scheme to compare integers with ($\lambda d \text {log} N +6d\lambda +3d$) modular multiplications (*d* is the length of inputs, $\lambda$ is set to 40–50). Blake and Kolesnikov (2004) uses the Paillier encryption scheme to solve the GT problem, the computation cost is $4d \text {log}N$ modular multiplications. Lin and Tzeng (2005) uses ($5d \text {log}p+4d-6$) modular multiplications (*p* is the modulus in the ElGamal encryption scheme) to determine $x>y$ or $x\le y$. Grigoriev and Shpilrain (2014) use a public encryption scheme to solve the Millionaires’ Problem and the computation cost is $(6\text {log}p+3d)$ modular multiplications. Maitra et al. (2015) solve the Millionaires’ problem with $(2d\text {log}p)$ modular multiplications.

In Protocol 2 and Protocol 3, we use the GM encryption scheme to encrypt the 0–1 encoding vector. The computation cost of the GM encryption scheme is three modular multiplications. So encrypting the vector needs 3*L* (*L* is the length of the 0–1 encoding vector) modular multiplications and decrypting $E_y'$ needs two modular multiplications. Therefore, the computation cost of Protocol 2 and Protocol 3 is ($2\times (3L+2))=(6L+4$) modular multiplications at most.

In Protocol 4, we do not use any public key encryption scheme, so we just needs five additions and eight multiplications. It is well known that simple operations can even be neglected compared with expensive public key encryption or decryption operations. In this sense, our new solution is much more efficient than the existing ones.

We compare our protocols with previous solutions in Table 1.

**Table 1:** Performance comparison

Protocol	Third party	Result	Data type	Round	Computation
Yao (1982)	No	$>, \le$	Integer	2	Exponential
Cachin (1999)	Yes	$>, =,<$	Integer	3	–
Fischlin (2001)	No	$>, \le$	Integer	2	$\lambda d \text {log} N +6d\lambda +3d$
Ioannidis and Grama (2003)	No	$\ge ,<$	Integer	d	–
Blake and Kolesnikov (2004)	No	$>,<$	Integer	2	$4d\text {log}N$
Lin and Tzeng (2005)	No	$>, \le$	Integer	2	$5d \text {log}p+4d-6$
Grigoriev and Shpilrain (2014)	No	$>, \le$	Integer	2	$6\text {log}p+3d$
Maitra et al. (2015)	No	$>, \le$	Integer	2	$2d\text {log}p$
Protocols 2, 3	No	$>, =,<$	Integer	2	$6L+4$
Protocol 4	No	$>, =,<$	Rational number	1	Negligible

*d* is the length of inputs, $\lambda$ is set to 40–50 in the Fischlin’s method (Fischlin 2001), *p* is the modulus in the ElGamal encryption scheme (ElGamal 1984), *N* is the modulo, *L* is the length of the 0–1 encoding vector in out work

Table 1 shows that our protocols have the following advantages:

1. Our protocols can determine whether $x>y, x<y$ or $x=y$, in one execution;
2. Our protocols can compare rational numbers in addition to integers;
3. Our protocols are more efficient than most of previous solutions in computational complexity.

## Conclusion

Solving a comparison problem privately is fundamental to SMC protocols, so the comparison problem needs to be computed more efficiently. In this paper, we propose protocols to compare integers and rational numbers privately. In Protocol 2 and Protocol 3, we construct a 0–1-vector encoding method to encode an integer into a vector, and use the GM encryption scheme to complete the protocol. In Protocol 4, we use the method of computing the area of a triangle to privately compare rational numbers by computing the sign of the area of a triangle. In comparison with previous solutions, our protocols are more efficient and easy to implement.

The comparison problem is a building block of SMC problems. If we can solve the problem efficiently, we will solve sorting problems and voting problems efficiently. Next we will solve geometric intersection problems and other SMC problems.
